# The relationship between exercise participation satisfaction, exercise commitment, and exercise adherence intention according to peer relationships among Korean school sports club participants

**DOI:** 10.3389/fpsyg.2025.1603098

**Published:** 2025-08-06

**Authors:** Tae Gyeom Jung, Myung Kyu Jung, Ji Hae Lee, Min Jun Kim

**Affiliations:** ^1^Department of Taekwondo, College of Taekwondo and Physical Education, Shinhan University, Uijeongbu-si, Gyeonggi, Republic of Korea; ^2^Department of Taekwondo, College of Social Science and Culture, Dongshin University, Naju-si, Jeollanam-do, Republic of Korea; ^3^Department of Golf Industry, College of Physical Education, Kyung Hee University, Yongin-si, Gyeonggi, Republic of Korea

**Keywords:** Korean school sports clubs, peer relationship, exercise participation satisfaction, exercise commitment, exercise adherence intention

## Abstract

**Introduction:**

This study investigated the structural relationships among peer relationships, exercise participation satisfaction, exercise commitment, and exercise adherence intention in the context of Korean adolescents participating in school sports clubs. The aim was to provide empirical insights that can inform strategies to promote sustained participation in physical activity.

**Methods:**

This study collected data from 245 middle school students in South Korea who were either currently participating in or had previous experience with school sports clubs, using a snowball sampling method. The data collection process was conducted with prior approval from the Institutional Review Board (IRB), and informed consent was obtained from both the participants and their legal guardians. The collected data were analyzed using frequency analysis, descriptive statistics, confirmatory factor analysis (CFA), reliability analysis, correlation analysis, and structural equation modeling (SEM).

**Results:**

The results indicated that peer relationships, exercise participation satisfaction, and exercise commitment significantly influenced exercise adherence intention. Peer relationships had a direct effect on both exercise participation satisfaction and exercise commitment, and an indirect effect on exercise adherence intention through these mediating variables.

**Discussion:**

These findings highlight the importance of developing intervention strategies that foster positive emotional experiences—particularly satisfaction and commitment—arising from peer relationships. Such strategies may be critical for encouraging long-term engagement in school-based sports programs among adolescents.

## 1 Introduction

As the value of individuality and personal time becomes increasingly significant, there is a growing societal interest in work-life balance. This heightened attention has spurred Korean teenagers, who often engage in fierce competition for entrance exams, to seek a balance between their studies and everyday lives (Kim and Yun, 2022). Physical activity is expected to be a pivotal factor in achieving a study-life balance among adolescents. Considering that leisure activities play a crucial role in enhancing quality of life (Brajša-Žganec et al., 2011), physical pursuits, especially sports participation, are particularly important. They serve as a counterbalance in Korea's entrance exam-focused educational environment, which often tilts the scale unfavorably between the students' lives and their academic commitments (Son and Kim, 2020).

Despite the recognized importance of physical activity, Korean teenagers' participation rate in such activity has been declining. Numerous studies indicate that most Korean teenagers prefer sedentary activities such as computer gaming and watching TV (Kim and Yun, 2022; Son and Kim, 2020; Yun and Kim, 2022). This trend intensified in the COVID-19 pandemic. Given that this shift can lead to physical and mental health issues beyond just impacting leisure time, it is crucial to devise strategies to promote physical activity in adolescents.

Efforts to promote physical activity in adolescents need to consider the intention to continue such activity and this factor has been investigated in various contexts related to exercise engagement. Exercise adherence (Dishman, 1988) is defined as regular and direct participation in physical activity. This includes metrics such as exercise frequency, intensity, duration, and overall physical activity (Choi, 2004; Dishman, 1988, 1994). Notably, exercise adherence is distinct from exercise participation. It emphasizes sustained engagement, making it more significant in terms of meaning and importance. Choi and Kim (2004) highlight that investigating exercise adherence could offer valuable insights into encouraging consistent participation in regular exercise and physical activity programs.

Prior research on exercise adherence has highlighted that positive emotions derived from physical activities, such as exercise participation satisfaction and commitment, can significantly influence an individual's intention to continue exercising. Here, “exercise participation satisfaction” is understood as the positive perception or emotion developed through the act of participating in exercise, viewed from the perspective of leisure satisfaction (Beard and Ragheb, 1980). By contrast, “exercise commitment” pertains to the hope, belief, and conviction acquired through engaging in physical activity that it is worth pursuing, combined with the desire for sustained participation (Kwon, 2011; Scanlan et al., 1993, 2016).

Previous research (Brickman, 1987; Cronin et al., 2000; Iso-Ahola, 1980; Park and Joo, 2019; Snyder and Spreitzer, 1973) has established that satisfaction and commitment are key predictors of exercise adherence. Empirically, studies by Park and Lee (2020) and Yang and Lee (2019) underscored that among Korean school sports club participants, satisfaction with exercise participation and commitment could serve as vital determinants of exercise adherence.

Peer relationships are regarded as a pivotal characteristic of adolescents. A “peer relationship” is defined as a systematic and ongoing dynamic interaction (Perry and Bussey, 1983; So and Cho, 2013) between individuals who share emotional bonds. This concept has been explored in the contexts of interpersonal relationships, friendships, and teenage bonds. Csikszentmihalyi and Larson (1986) observed that in contrast to childhood, peer relationships in adolescence can significantly influence an individual's emotional state across various situations, especially as the time spent with peers increases. Supporting this perspective, Rain et al. (1991) posit that satisfaction derived from one life domain could spill over into another. Earlier research (Burleson et al., 1994; Goldsmith, 2011; Goldsmith and Albrecht, 2011) had indicated that an individual's emotional response to physical activity might shift based on social relationships.

Drawing on previous studies that demonstrate the influence of peer relationships on satisfaction (Ash and Huebner, 1998; Choi and Kim, 2017; Lee and Jun, 2021; Purpora and Blegen, 2015), commitment (Song and Lee, 2014; Yune and Kang, 2012), adherence, and dropout intentions (Chung, 2004; Heo et al., 2016; Kovács, 2025; So and Cho, 2013), these elements may be expected to share a structural relationship. Furthermore, peer relationships may be projected to serve as predictors of exercise participation, satisfaction, commitment, and adherence intention.

However, prior studies have not comprehensively examined the structural interrelationships among peer relationships, exercise participation satisfaction, exercise commitment, and exercise adherence intention. In particular, empirical research focusing on the relationships among these variables in the context of adolescents participating in school sports clubs remains notably scarce. As a result, empirical understanding of the structural relationships among these variables within this specific population remains limited.

Therefore, this study aimed to explore the structural relationships among peer relationships, exercise participation satisfaction, exercise commitment, and exercise adherence intention among participants in school sports clubs by utilizing a structural equation model. “Sports clubs” refer to a regular part of the curriculum introduced across all Korean middle schools in 2007 to address the decline in physical activity and growing disinterest in leisure activities among teenagers, thereby encouraging autonomous sports activities for students (Kim, 2018). Considering the significant time constraints that Korean teenagers face in physical activity, primarily due to the education system's focus on college entrance exams, exploring these relationships within the context of school sports clubs has substantial relevance, is novel, and offers the prospect of useful findings.

This study explored the structural relationships between peer relationships, exercise participation satisfaction, exercise commitment, and exercise adherence intention in the context of school sports club participants with the aim to offer valuable insights into devising effective strategies tailored to promote consistent exercise habits, taking into account the unique situations and traits of Korean adolescents.

Past research indicates that factors such as peer relationships, exercise participation satisfaction, and exercise commitment positively influence exercise adherence intention (Ash and Huebner, 1998; Choi and Kim, 2017; Kovács, 2025; Lee and Jun, 2021; Parker and Asher, 1987; Purpora and Blegen, 2015; So and Cho, 2013; Song and Lee, 2014; Yune and Kang, 2012). Notably, peer relationships have been shown to bolster both satisfaction and exercise commitment (Brickman, 1987; Cronin et al., 2000; Iso-Ahola, 1980; Park and Joo, 2019; Park and Lee, 2020; Snyder and Spreitzer, 1973; Yang and Lee, 2019). Thus, it was anticipated that similar relationships would emerge among participants of school sports clubs in relation to peer interactions, satisfaction with exercise participation, commitment, and the intention to continue exercising. Seven hypotheses were formulated.

H1: Peer relationships among school sports club participants would have a significant effect on exercise participation satisfaction.
H2: Peer relationships among school sports club participants would have a significant effect on exercise commitment.
H3: Peer relationships among school sports club participants would have a significant effect on exercise adherence intentions.
H4: Exercise participation satisfaction in school sports club participants would have a significant effect on exercise adherence intention.
H5: Exercise commitment of school sports participants would have a significant effect on their exercise adherence intention.

Drawing from prior research, it was anticipated that satisfaction and commitment with exercise participation would significantly affect the relationship between peer relationships and exercise adherence intention among school sports club participants. Peer relationships foster positive emotions, such as satisfaction with exercise participation and commitment, which in turn can increase the propensity for sustained exercise adherence. This understanding is supported by the findings from Kim (1989), which highlighted the mediating roles of adherence and satisfaction in the relationship between social support from elementary school sports club leaders and the intention to adhere to exercise.

However, there is a notable gap in research probing the structural relationship between satisfaction, commitment, and intention, especially when centered on peer relationships. Furthermore, empirical studies of school sports club participants are scarce. To explore this relationship and related mediating effects among these types of participants, the following hypotheses were formulated.

H6: Satisfaction with exercise participation would have a mediating effect on the relationship between the peer relationships of school sports club participants and exercise adherence intention.
H7: Exercise commitment would mediate the relationship between the peer relationships of school sports club participants and exercise adherence intention.

## 2 Methods

### 2.1 Participants and procedure

The data for this study were collected December 2024 using snowball sampling. This study was approved by the Institutional Review Board of Dongshin University (IRB No: 1040708-202410-SB-031). The participants were teenagers who were either actively enrolled in school sports clubs or participated in after-school activities at middle schools in South Korea.

Data were collected through an online survey. Prior to distribution, schools with the potential to cooperate were identified and reviewed. With the assistance of teachers from participating schools, the survey was administered following the acquisition of informed consent from both the legal guardians and the student participants through an online consent procedure. In addition, prior to data collection, the minimum required sample size for this study was calculated using G^*^Power 3.1 (*f*^2^ = 0.15, α = 0.05, power = 0.95, with three predictors), which indicated that at least 119 participants were needed. However, considering the complexity of the structural equation model (SEM), a larger sample of 245 participants was collected to ensure model stability and validity (Kline, 2016).

The survey breakdown of the collected samples was as follows. Of the participants, 158 were male (64.5%) and 87 were female (35.5%). Based on grade level, 62 were first graders (25.3%), 94 were second graders (38.4%), and 89 were third graders (36.3%).

### 2.2 Measurement

The questionnaire consisted of four primary constructs: peer relationships, satisfaction with exercise participation, exercise commitment, and exercise adherence intention. Validated existing instruments were adapted to fit the specific context of this study. All items were rated on a 5-point Likert scale ranging from 1 (strongly disagree) to 5 (strongly agree).

The peer relationship scale was originally developed by Kim (1989), based on earlier instruments employed by Park and Kim (2007), and was subsequently revised and adapted for use with physical activity participants by Park (2016) and Lee (2019). Through a content validity assessment (e.g., expert panel evaluation of item appropriateness for the target population) and a variable refinement process (e.g., deletion of items based on squared multiple correlations [SMC] and modification indices [MI]), the scale was reorganized into four subfactors: Presence of Friendship (5 items), Continuation of Relationship (4 items), Friend Adaptation (4 items), and Co-living (3 items).

The exercise participation satisfaction scale was adapted from the Leisure Satisfaction Scale developed by Beard and Ragheb (1980) and was revised to reflect the context of physical activity, drawing on previous studies (Kang and Lee, 1997; Kim, 2000). Following content validation and item refinement based on SMC and MI values, the scale was restructured into four subfactors: Intrapersonal Psychological Satisfaction (5 items), Social Satisfaction (4 items), Physical Satisfaction (4 items), and Environmental Satisfaction (4 items).

The exercise commitment scale was derived from the sport commitment model proposed by Scanlan et al. (1993) and was modified to reflect the Korean sociocultural context by Jung (1997). As with the other scales, content validity was established through expert review, and low-performing items were removed based on SMC and MI indices. The final scale comprised two subfactors: Cognitive Commitment (4 items) and Behavioral Commitment (4 items).

Lastly, the exercise adherence intention scale was developed based on a Korean-specific instrument constructed by Choi (2005), which was grounded in the theoretical frameworks and empirical findings of Fishbein and Ajzen (1980) and Stebbins (1982, 2001). Following expert validation and a refinement process using SMC and MI values, the scale was reorganized into five subfactors: Exercise Ability (3 items), Exercise Habit (3 items), Exercise Environment (4 items), Exercise Interest (3 items), and Exercise Friend (3 items).

### 2.3 Data analysis

A total of 245 questionnaire responses were used in the final validation sample. For data analysis, IBM SPSS ver. 28.0 software was employed for frequency, descriptive, reliability, and correlation analyses. Confirmatory factor analysis (CFA) and structural equation modeling (SEM) were performed using IBM AMOS ver. 21.0 software. The significance of specific indirect effects was assessed through bootstrapping (ML, Bias-corrected confidence intervals = 95%, Perform bootstrap = 2000), considering the characteristics of the research model.

## 3 Results

Data normality was assessed using skewness and kurtosis values. All values obtained conformed to the recommended criteria (West et al., 1995). Specifically, skewness values ranged from −0.825 to 0.194, while kurtosis values were between −0.607 and 0.084. Given that the acceptable limits for skewness and kurtosis are ±2 and ±4, respectively, the data met the normality assumption. The relevant details are presented in Table 1.

**Table 1:** Results of means, standard deviations, Skewness, Kurtosis, and bivariate correlations.

	**1**	**2**	**3**	**4**	**5**	**6**	**7**	**8**	**9**	**10**	**11**	**12**	**13**	**14**	**15**
1	1														
2	0.648^**^	1													
3	0.684^**^	0.611^**^	1												
4	0.644^**^	0.645^**^	0.619^**^	1											
5	0.561^**^	0.562^**^	0.522^**^	0.480^**^	1										
6	0.491^**^	0.444^**^	0.472^**^	0.405^**^	0.475^**^	1									
7	0.526^**^	0.516^**^	0.484^**^	0.459^**^	0.540^**^	0.558^**^	1								
8	0.526^**^	0.546^**^	0.480^**^	0.454^**^	0.569^**^	0.620^**^	0.622^**^	1							
9	0.514^**^	0.413^**^	0.414^**^	0.380^**^	0.388^**^	0.446^**^	0.453^**^	0.518^**^	1						
10	0.454^**^	0.473^**^	0.377^**^	0.332^**^	0.516^**^	0.369^**^	0.486^**^	0.417^**^	0.461^**^	1					
11	0.681^**^	0.664^**^	0.614^**^	0.594^**^	0.667^**^	0.492^**^	0.593^**^	0.624^**^	0.459^**^	0.589^**^	1				
12	0.571^**^	0.529^**^	0.505^**^	0.501^**^	0.561^**^	0.470^**^	0.533^**^	0.501^**^	0.452^**^	0.452^**^	0.642^**^	1			
13	0.604^**^	0.557^**^	0.602^**^	0.509^**^	0.479^**^	0.471^**^	0.481^**^	0.528^**^	0.483^**^	0.399^**^	0.601^**^	0.588^**^	1		
14	0.579^**^	0.533^**^	0.525^**^	0.527^**^	0.543^**^	0.497^**^	0.489^**^	0.567^**^	0.514^**^	0.480^**^	0.640^**^	0.543^**^	0.482^**^	1	
15	0.647^**^	0.591^**^	0.572^**^	0.584^**^	0.563^**^	0.488^**^	0.522^**^	0.541^**^	0.545^**^	0.468^**^	0.635^**^	0.553^**^	0.669^**^	0.667^**^	1
M	3.76	3.64	3.64	3.65	3.56	3.86	3.59	3.75	3.47	3.29	3.68	3.57	3.60	3.27	3.52
SD	0.98	0.88	0.98	0.94	1.11	1.12	1.10	1.20	1.06	0.98	0.92	1.02	0.99	1.01	0.99
S	−0.66	−0.38	−0.68	−0.51	−0.60	−0.83	−0.59	−0.71	−0.43	0.19	−0.38	−0.45	−0.68	−0.15	−0.50
K	−0.07	−0.54	0.08	−0.33	−0.38	−0.34	−0.40	−0.61	−0.53	−0.41	−0.46	−0.50	0.08	−0.56	−0.23

1, presence of friendship; 2, continuation of relationship; 3, friend adaptation; 4, co-living; 5, intrapersonal psychological satisfaction; 6, social satisfaction; 7, physical satisfaction; 8, environmental satisfaction; 9, cognitive commitment; 10, behavioral commitment; 11, exercise ability; 12, exercise habit; 13, exercise environment; 14, exercise interest; 15, exercise friend;

** p < 0.01.

### 3.1 Measurement model

To examine the validity and reliability of the measurement tool, we conducted a confirmatory factor analysis of the measurement model and a reliability analysis using Cronbach's α coefficient. As a detailed explanation of the analysis results, first, results satisfying the model fit indices proposed by the American Psychological Association (APA) were detected (*x*^2^/DF= 1.521, CFI = 0.950, TLI = 0.945, SRMR = 0.037, RMSEA = 0.046). Subsequent additional analysis detected measures of AVE above 0.5 (from 0.567 to 0.884), CR above 0.7 (from 0.797 to 0.968), and α above 0.7 (from 0.838 to 0.976), and based on these, it was inferred that convergent validity and reliability were satisfied (Bae, 2017; Garrido et al., 2016; Jung et al., 2024). Moreover, the AVE index (0.567) exceeded the squared correlation coefficient value (0.468) of the maximum correlation (0.684), indicating satisfactory discriminant validity (Anderson and Gerbing, 1988; Bae, 2017; Fornell and Larcker, 1981; Jung et al., 2024). The relevant details are presented in Tables 1, 2.

**Table 2:** Results of the measurement model analysis.

**Construct and item**	**λ**	**a**	**AVE**	**C.R**.
**Peer relationship (PR)**				
Presence of friendship (PF)		0.939	0.724	0.929
PF 1	0.883			
PF 2	0.824			
PF 3	0.885			
PF 4	0.868			
PF 6	0.889			
Continuation of relationship (CR)		0.892	0.669	0.889
CR 7	0.861			
CR 9	0.755			
CR 10	0.806			
CR 11	0.859			
Friend adaptation (FA)		0.909	0.674	0.892
FA 13	0.841			
FA 14	0.868			
FA 15	0.837			
FA 17	0.837			
Co-living (CL)		0.853	0.637	0.840
CL18	0.739			
CL 19	0.841			
CL 20	0.867			
**Exercise participation satisfaction (EPS)**				
Intrapersonal psychological satisfaction (IP)		0.972	0.836	0.962
IP 1	0.927			
IP 2	0.930			
IP 3	0.961			
IP 4	0.945			
IP 5	0.909			
Social satisfaction (SS)		0.976	0.884	0.968
SS 7	0.946			
SS 8	0.972			
SS 9	0.954			
SS 10	0.945			
Physical satisfaction (PS)		0.951	0.779	0.934
PS 11	0.902			
PS 13	0.896			
PS 14	0.919			
PS 15	0.925			
Environmental satisfaction (ES)		0.975	0.866	0.963
ES 16	0.917			
ES 17	0.959			
ES 18	0.975			
ES 19	0.958			
**Exercise commitment (EC)**				
Cognitive commitment (CC)		0.917	0.696	0.900
CC 3	0.981			
CC 4	0.747			
CC 5	0.768			
CC 6	0.982			
Behavioral commitment (BC)		0.927	0.731	0.916
BC 8	0.872			
BC 9	0.884			
BC 11	0.863			
BC 12	0.869			
**Exercise adherence intention (EA)**				
Exercise ability (ExA)		0.872	0.684	0.866
ExA 1	0.863			
ExA 2	0.818			
ExA 4	0.822			
Exercise habit (ExH)		0.881	0.659	0.853
ExH 5	0.796			
ExH 6	0.900			
ExH 7	0.846			
Exercise environment (ExE)		0.917	0.697	0.902
ExE 8	0.834			
ExE 9	0.840			
ExE 10	0.829			
ExE 11	0.924			
Exercise interest (ExI)		0.838	0.567	0.797
ExI 12	0.773			
ExI 13	0.803			
ExI 14	0.817			
Exercise friend (ExF)		0.862	0.650	0.846
ExF15	0.703			
ExF 16	0.865			
ExF 17	0.916			

1: Model fit: x^2^/DF = 1.521(x^2^ = 2181.633, DF = 1434), comparative fit index (CFI) = 0.950, Tucker-Lewis index (TLI) = 0.945, standardized root mean square residual (SRMR) = 0.037, root mean square error of approximation (RMSEA) = 0.046.

2: λ = Factor loading; a = Cronbach's alpha; CR = Construct reliability; AVE = Average variance extracted.

### 3.2 Hypotheses testing

After assessing the fit of the structural equation model prior to hypothesis testing, the results (*x*^2^/DF= 2.051, CFI = 0.961, TLI = 0.951, SRMR = 0.042, RMSEA = 0.066) indicated a suitable fit for the hypothesis equation in this study (Bae, 2017; Garrido et al., 2016; Jung et al., 2024). The relevant details are presented in Table 3.

**Table 3:** Results of structural equation modeling.

	**Path of latent variables**	**Direct effect**	**Indirect effect**	***p*-value**	**Hypothesis testing**
H1	PR -> EPS	0.855 (0.852)^***^		<0.001	Supported
H2	PR -> EC	0.733 (0.834)^***^		<0.001	Supported
H3	PR -> EA	0.391 (0.416)^**^		0.005	Supported
H4	EPS -> EA	0.292 (0.311)^***^		<0.001	Supported
H5	EC -> EA	0.346 (0.325)^*^		0.011	Supported
H6	PR -> EPS -> EA		0.249 (0.265)^*^	0.016	Supported
H7	PR -> EC -> EA		0.255 (0.271)^**^	0.006	Supported

1: Model fit: x^2^/DF = 2.051(x^2^ = 174.365, DF = 85), comparative fit index (CFI) = 0.961, Tucker-Lewis index (TLI) = 0.951, standardized root mean square residual (SRMR) = 0.042, root mean square error of approximation (RMSEA) = 0.066; ^***^p < 0.001; ^**^p < 0.01, ^*^p < 0.05.

2: PR, Peer Relationship; EPS, Exercise Participation Satisfaction; EC, Exercise Commitment; EA, Exercise Adherence.

3: Bootstrapping = ML, Bias-corrected confidence intervals = 95%, Perform bootstrap = 2,000.

**Figure 1:**
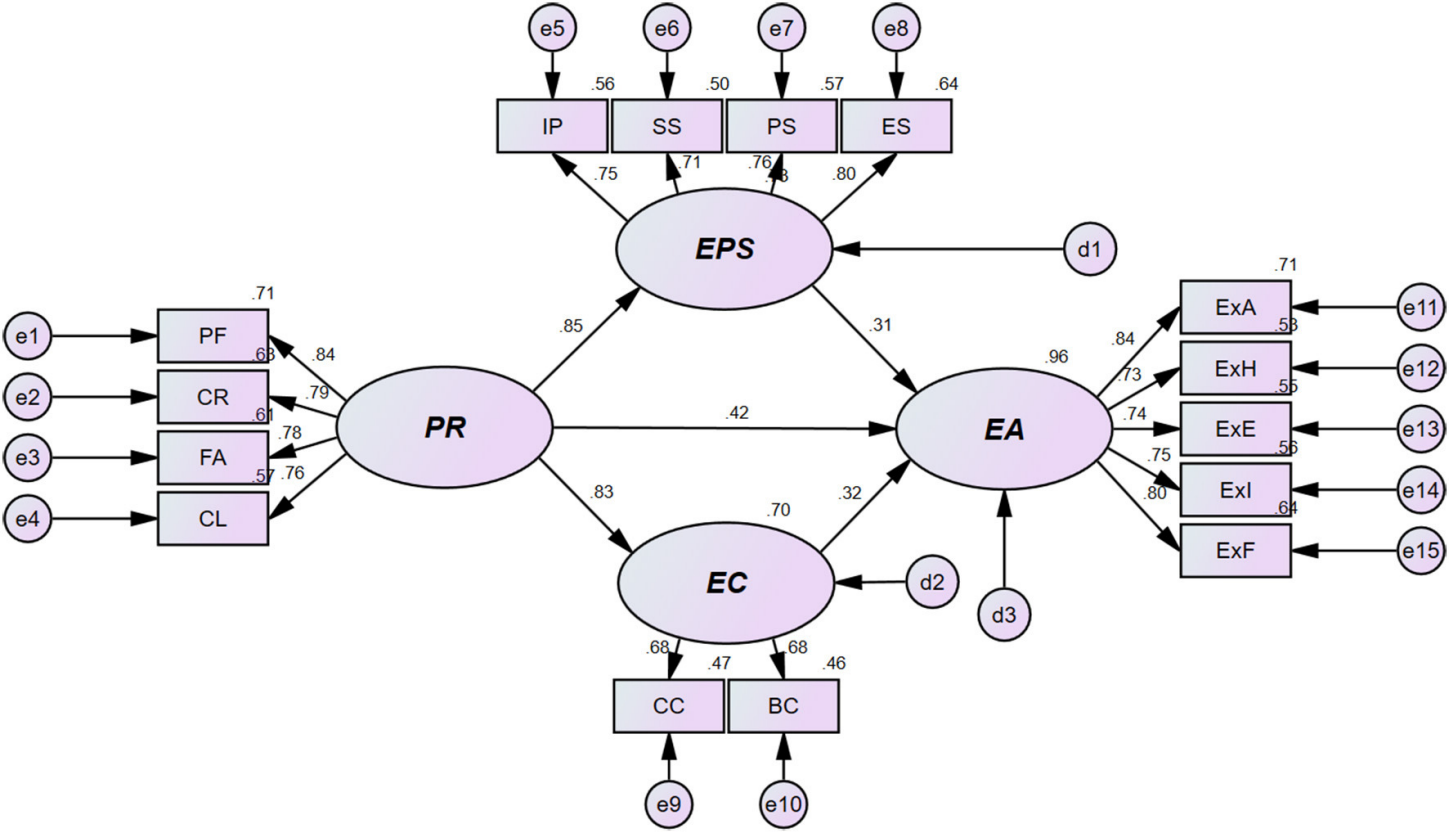
Results of the structural equation modeling analysis.

Based on the suitability of the estimated research model, the verification results of the research hypotheses proposed to investigate the structural relationship among peer relations, exercise participation satisfaction, exercise commitment, and exercise continuation were as follows. The relevant details are presented in Table 3 and Figure 1. First, the statistical significance of peer relationships in relation to exercise participation satisfaction and exercise commitment was verified, and therefore, H1 (b = 0.855, β = 0.852, *p* < 0.001) and H2 (b = 0.733, β = 0.834, *p* < 0.001) were confirmed. Second, the statistical significance of peer relationships, exercise participation satisfaction, and exercise commitment in relation to exercise continuation intention was verified; thus, H3 (b = 0.391, β = 0.416, *p* < 0.01), H4 (b = 0.292, β = 0.311, *p* < 0.001), and H5 (b = 0.346, β = 0.325, *p* < 0.05) were confirmed.

Given that our research model comprises a parallel multiple-mediator model structure, it was essential to estimate specific indirect effects. In multi-mediator models with several indirect effects, a “specific indirect effect” pertains to the indirect effect associated with a particular variable. This estimation is significant because the indirect effect typically calculated in AMOS represents the total indirect effect.

**Figure 2:**
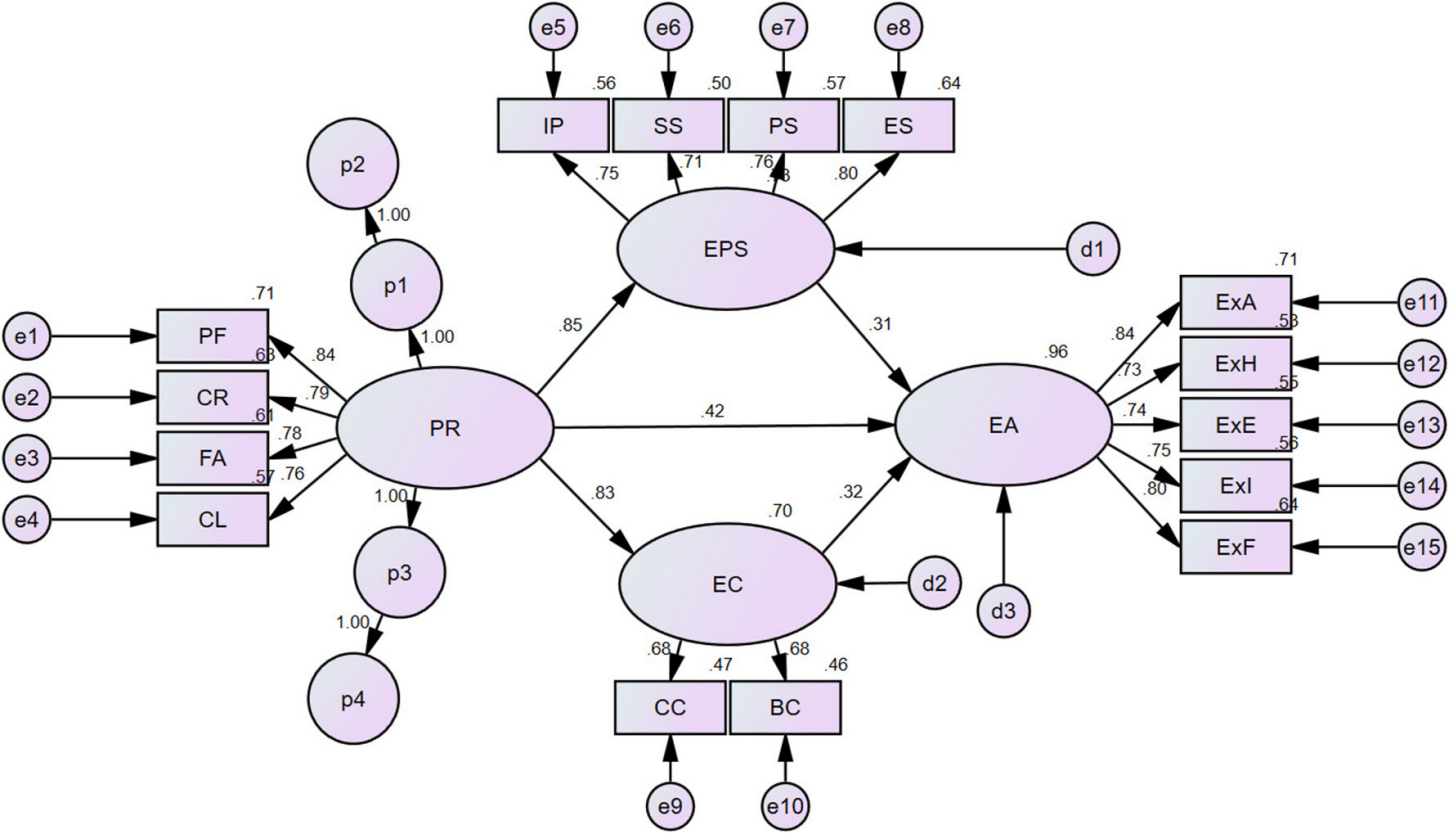
Results of the structural equation model analysis using Phantom variables structural equation model analysis.

Using the IBM AMOS software, the phantom variable technique was employed to estimate specific indirect effects and evaluate their significance. The detailed outcomes of the analysis derived from the phantom variable estimation are outlined in Table 3 and Figure 2. First, regarding the relationship between peer relationships and exercise adherence intention, the specific indirect effects of satisfaction with exercise participation and commitment were b(β) = 0.249 (0.265) and b(β) = 0.255 (0.271), respectively. Both H6 (*p* < 0.05) and H7 (*p* < 0.01) were confirmed, and their significance was substantiated using non-parametric bootstrapping.

## 4 Discussion

In this study, we use a structural equation model to explore the direct effects of peer relationships, satisfaction with exercise participation, exercise commitment, and exercise adherence intention on participants in school sports clubs. Furthermore, we examine the mediating role of satisfaction with exercise participation and commitment in the relationship between peer relationships and exercise adherence intention.

Our findings reveal that peer relationships, exercise participation satisfaction, and exercise commitment positively influenced adherence intention among school sports club participants, confirming H3, H4, and H5. These results align with those of prior research (Ash and Huebner, 1998; Choi and Kim, 2017; Kovács, 2025; Lee and Jun, 2021; Parker and Asher, 1987; Purpora and Blegen, 2015; So and Cho, 2013; Song and Lee, 2014; Yune and Kang, 2012), emphasizing the significance of these factors as determinants of adherence intention. Peer relationships, satisfaction with exercise participation, and commitment to exercise were found to be crucial psychological factors that foster regular and sustained participation in school sports club activities. Their impact on exercise adherence is further underscored in the current literature, which highlights the vital roles of these psychological factors. Positive experiences and emotions stemming from peer relationships, satisfaction, and commitment during exercise activities can directly enhance adherence.

The study findings also indicate that peer relationships positively influenced not only the exercise adherence of school sports club participants, but also their participation satisfaction and exercise commitment, confirming H1 and H2. This underscores the importance of emotional social support among these participants as a pivotal psychological factor that contributes to their satisfaction with exercise participation and commitment. These findings align with prior research in Korea that explored the interplay between these variables (Choi and Kim, 2017; Lee and Jun, 2021; Yune and Kang, 2012).

The impact of peer relationships on satisfaction and commitment to exercise participation can be attributed to the emotional dimension of satisfaction. As Rain et al. (1991) posit, satisfaction in relation to an individual's experiences can catalyze various positive outcomes. This suggests that satisfaction derived from school sports club activities and an individual's psychological state in diverse scenarios are interrelated, rather than isolated phenomena. Moreover, peer relationships can engender myriad emotions in school sports clubs. For instance, contentment with peers involved in physical activities becomes instrumental. Such emotions can serve as significant predictors of exercise participation satisfaction, and commitment.

Furthermore, given the significant relationships established between peer relationships, exercise participation satisfaction, exercise commitment, and exercise adherence intention, both exercise participation satisfaction and commitment were expected to mediate the link between peer relationships and exercise adherence intention. This mediating effect was evident in both exercise satisfaction and exercise engagement (H6 and H7). This suggests that peer relationships can foster positive emotions such as satisfaction and commitment to participation in sports as a form of intrinsic motivation, and that positive emotions in turn amplify exercise adherence intentions. And these findings ultimately highlight the role of peer relationships as a powerful psychological driver of continued participation in school sports clubs.

In the Korean educational landscape, where college entrance examinations hold significant weight (Son and Kim, 2020), teenagers often struggle to maintain a sometimes precarious balance between life and academic requirements. Physical activity is crucial in such environments. Specifically, school sports club activities stand out as a hallmark of physical engagement among Korean adolescents and play a crucial role in enhancing their overall quality of life (Brajša-Žganec et al., 2011).

The significance of factors such as peer relationships, satisfaction with exercise participation, and exercise commitment has not been previously understood within the overall framework of the lives of such adolescents. However, the findings of this study indicate that these are the key determinants in sustaining youth engagement in physical activities, particularly in school sports clubs. Fostering strong peer relationships can serve as an effective strategy to motivate students to remain active participants in school sports clubs.

It is imperative to recognize the significance of peer relationships, satisfaction with exercise participation, and exercise commitment in fostering sustained engagement among school sports club participants. Such recognition can inform the development of meaningful intervention strategies that promote positive emotional experiences centered on peer relationships. These relationships, often cultivated through close interactions and social support (Buhrmester and Furman, 1990; Bum and Jeon, 2016; Furman and Buhrmester, 1992), can facilitate spontaneous and voluntary participation. In this regard, various teamwork activities implemented prior to sports participation in school sports clubs are expected to foster and strengthen peer relationships among participants.

## 5 Conclusion

This study aimed to provide foundational data for developing effective strategies to enhance exercise adherence intentions tailored to the characteristics and circumstances of Korean adolescents by identifying the structural relationships among peer relationships, exercise participation satisfaction, exercise commitment, and exercise adherence intention among school sports club participants. Through hypothesis testing, the study confirmed that these variables are significantly related both directly and indirectly. Based on these findings, the study emphasizes the importance of recognizing the roles of peer relationships, participation satisfaction, and commitment in promoting continued exercise participation in school sports club settings, highlighting the need to develop intervention strategies that foster positive emotions, particularly through peer relationships.

However, this study has several limitations. It employed a non-probability sampling method and focused exclusively on Korean adolescents, thereby limiting the generalizability of the findings to broader populations. To enhance external validity, future research should adopt probability sampling techniques and include a more diverse range of demographic characteristics—such as gender, type of sport participation, and cultural background. Furthermore, since this study utilized a cross-sectional design based on data collected at a single point in time, longitudinal research is warranted to examine how peer relationships, exercise participation satisfaction, commitment, and exercise adherence intentions develop and interact over time.

## Data Availability

The original contributions presented in the study are included in the article/supplementary material, further inquiries can be directed to the corresponding author.

## Nomenclature

Peer relationship: peer relationship refers to a systematic and continuous dynamic relationship.

Excise participation satisfaction: excise participation satisfaction refers to the positive perception or emotion formed or acquired in the process of participating in exercise from the perspective of leisure satisfaction.

Exercise commitment: exercise commitment refers to the hope, belief, and belief gained through exercise participation and the desire to continuously participate in exercise.

Exercise adherence: Exercise adherence is a particular individual participates in exercise directly and performs the activity regularly.
